# Sample Entropy Identifies Differences in Spontaneous Leg Movement Behavior between Infants with Typical Development and Infants at Risk of Developmental Delay

**DOI:** 10.3390/technologies5030055

**Published:** 2017-09-02

**Authors:** Beth A. Smith, Douglas L. Vanderbilt, Bryon Applequist, Anastasia Kyvelidou

**Affiliations:** 1 Division of Biokinesiology and Physical Therapy, University of Southern California, Los Angeles, CA 90089-9006, USA; 2 Department of Pediatrics, Division of General Pediatrics, Keck School of Medicine, University of Southern California, Los Angeles, CA 90089-9234, USA; 3 Department of Exercise Science and Sport, University of Scranton, Scranton, PA 18510, USA; 4 Department of Physical Therapy, Creighton University, Omaha, NE 68178, USA

**Keywords:** wearable sensors, infants, leg movement, movement system, sample entropy, variability

## Abstract

We are interested in using wearable sensor data to analyze detailed characteristics of movement, such as repeatability and variability of movement patterns, over days and months to accurately capture real-world infant behavior. The purpose of this study was to explore Sample Entropy (SampEn) from wearable sensor data as a measure of variability of spontaneous infant leg movement and as a potential marker of the development of neuromotor control. We hypothesized that infants at risk (AR) of developmental delay would present significantly lower SampEn values than infants with typical development (TD). Participants were 11 infants with TD and 20 infants AR. We calculated SampEn from 1–4 periods of data of 7200 samples in length when the infants were actively playing across the day. The infants AR demonstrated smaller SampEn values (median 0.21) than the infants with TD (median 1.20). Lower values of SampEn indicate more similarity in patterns across time, and may indicate more repetitive, less exploratory behavior in infants AR compared to infants with TD. In future studies, we would like to expand to analyze longer periods of wearable sensor data and/or determine how to optimally sample representative periods across days and months.

## 1. Introduction

Studies of infant leg movements tend to quantify infants’ behavior from seconds [1] to minutes [2,3], up to an hour or two [4–6]. Despite the great knowledge gathered from these studies, it has recently been argued that to further advance the field, we must use new technologies to sample development for a minimum of 24 h per sample in order to overcome the effects of circadian rhythms, behavioral context, environmental stimuli, mood and motivation, among others [7]. Newer technologies, namely accelerometers, allow for longer-term data collection and analysis of infants behavior over 24 or even 48 continuous hours [8–10]. For example, researchers quantified leg activity of infants with and without Down syndrome over 48 h at 3, 4, 5, and 6 months of age as low or high intensity activity. Results showed that infants with Down syndrome spent more time producing low intensity activity during the day than the infants with typical development. The results also indicated that infants (both with and without Down syndrome) who spent more time producing low intensity activity during the day had later onsets of walking [9]. Although these studies using accelerometers to quantify low and high intensity movement are useful and allow continuous analysis over 24 or 48 h, they do not allow analysis of the more detailed characteristics of movement, such as repeatability and variability of movement over time.

This natural variability has been widely neglected in the past, and was considered random error or noise within the system [11,12]. In contrast, rather than being a negative feature, variability reflects important information for the maintenance of the health of the system. Specifically, the increased utilization of temporal measures of variability for motor related deficits, revealed altered coordination in the motor control system in infants with cerebral palsy [13,14], elderly at risk of falling [15], patients with multiple sclerosis [16], Parkinson’s disease [17], peripheral arterial disease [18], and others [19,20]. Sample Entropy (SampEn) has been proposed as a measure of signal regularity with the potential to inform us about patterns of infants’ spontaneous leg movements over time. SampEn is advantageous in comparison to Approximate Entropy as it is more robust with shorter data sets and presents fewer issues with consistency as parameter selection changes. Entropy measures have been used widely in the past to determine the probability that similar patterns of organization in movement data will not be followed in time by similar organizations in infants while sitting [13,21]. Entropy measures from acceleration data have been used from sensors placed on the trunk and foot in patients with multiple sclerosis and healthy young and elderly participants [22–25]. This analysis has added considerable information on the organization of movement in these populations and offered the opportunity to use sensors that do not restrict movement and allow collections outside the laboratory. However, the methodology of SampEn has not been used to identify the variability present in the spontaneous infant leg movements measured by movement sensors and accelerometry data.

Thus, the purpose of this study was to explore SampEn from wearable sensor data as a measure of variability of spontaneous infant leg movement and as a potential marker of the development of neuromotor control. Since we explored SampEn values from wearable sensor data from both infants with typical development (TD) and at risk (AR) of developmental delay, we hypothesized that infants AR would present significantly lower SampEn values than infants with TD.

## 2. Materials and Methods

### 2.1. Participants

Data were collected from 31 infants, 11 infants with TD and 20 infants AR. Infants were recruited by word of mouth, at Children’s Hospital of Los Angeles High Risk Follow-Up Clinic (a specialty interdisciplinary clinic for Neonatal Intensive Care Unit graduates), and at local primary care and early intervention provider locations. Participant characteristics are summarized in Table 1. Infants AR were defined as at risk in accordance with the definition set forth by the state of California (level medical risk criteria). Briefly, infants are identified as at risk of developmental disability according to known, population-based criteria including preterm birth, complications at or after birth, etc. The definition is used to define at risk of developmental delay and eligible for state administered early intervention and can be found, in full, in the reference [26]. This represents a heterogeneous AR group, with varying levels of developmental delay and complications, as followed in the clinical setting. It is anticipated that around half of them will have a diagnosis of developmental delay at 24 months of age. Infants with TD were from singleton, full-term pregnancies with scores above the 5th percentile on Alberta Infant Motor Scale at each visit [27].

### 2.2. Procedures

**Figure 1:**
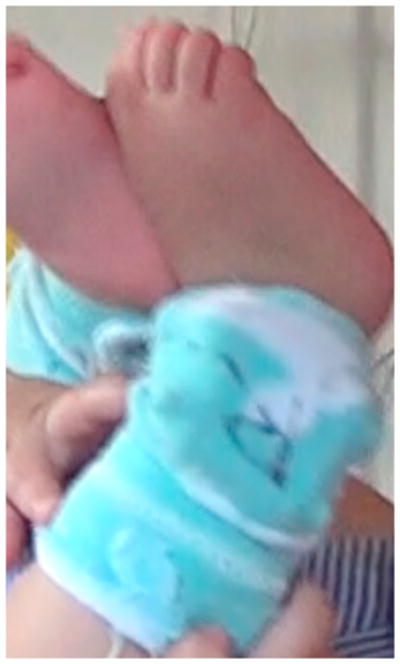
Infant wearing sensors on both ankles, inserted into custom-made garments with internal pockets to hold sensors.

This research was approved by the Institutional Review Boards of the University of Southern California or by Oregon Health & Science University. A parent or legal guardian signed an informed consent form prior to their infant’s participation. At each visit, we administered the Alberta Infant Motor Scale to quantify motor development status [27] and measured weight, length, and head circumference. We placed inertial movement sensors (Opals, APDM, Inc., Portland, OR, USA) on each leg using knee socks or legwarmers with pockets (see Figure 1). They collected actively synchronized tri-axial accelerometer and gyroscope data at 20 samples per second; an example of sensor data can be found in a previous publication [28]. We chose the sampling frequency at 20 Hz based on battery life and the fact that the power spectra analysis of infant leg movement revealed that 99% percent of the signal was below 2 Hz. Data were stored on each sensors’ internal memory, and downloaded following the collection. The visit always took place in the morning, and the sensors remained in place until bedtime, 8–13 h later, when a caregiver removed them. They were instructed to go about their normal activities throughout the day, keeping track of position and activity in a written log. They were asked to document a new entry each time the activity changed, as well as the start and end times. In addition, during the morning visit, spontaneous movement video data were recorded for 5 min, while the infant wore the sensors. Videos were obtained to provide gold standard observation of leg movements and will not be further discussed in this paper.

### 2.3. Data Analyses

We used the procedure of surrogation to validate whether movement sensor data of spontaneous leg movement in infants with TD and AR was deterministic (has order) or stochastic (random) in nature. Surrogate data sets were generated for all original time series. The surrogates were produced from the original data, but the deterministic structure from the original data set was removed, generating a random equivalent with the same mean, variance, and power spectra as the original. Original data significantly differed from surrogate (20 surrogates, 7200 data points when sensors on infant). This procedure allowed us to verify the determinism in the data and proceed with the calculation of SampEn.

SampEn was calculated using the algorithm of Richman and Moorman (2000) implemented in MATLAB (Mathworks, Natick, MA, USA) [29]. SampEn was defined as the negative logarithm for conditional properties that a series of data points within a certain distance, m, would be repeated within the distance m + 1. Two parameters must be set before the calculation of SampEn: (a) the relative tolerance limit (r), which is this number times the standard deviation of the sensor data; and (b) the vector length (m). For the present study, m was set at 2 and r at 0.2.

We calculated SampEn from 1–4 periods of data, 7200 samples in length, when the infants were actively playing per the activity log. Activity log entries reported, for example, “10:00–10:20 a.m., on back under infant gym” or “11:00–11:15 a.m., on floor crawling around, playing”. Data were selected by matching the time of the activity log to the time of the sensor data and were visually inspected to confirm that movements were occurring. The median value of these samples was entered into further analyses as their SampEn value. Data from the left leg were included in statistical analyses as there was not a difference between the right and left leg data.

### 2.4. Statistical Analyses

We used non-parametric Mann–Whitney tests to look for group differences in age (chronological or adjusted for prematurity, in months), Alberta Infant Motor Scale, and SampEn. Alpha level of significance was set at 0.05 and all tests were performed using SPSS software (Version 22, IBM Corporation, Armonk, NY, USA).

## 3. Results

**Figure 2:**
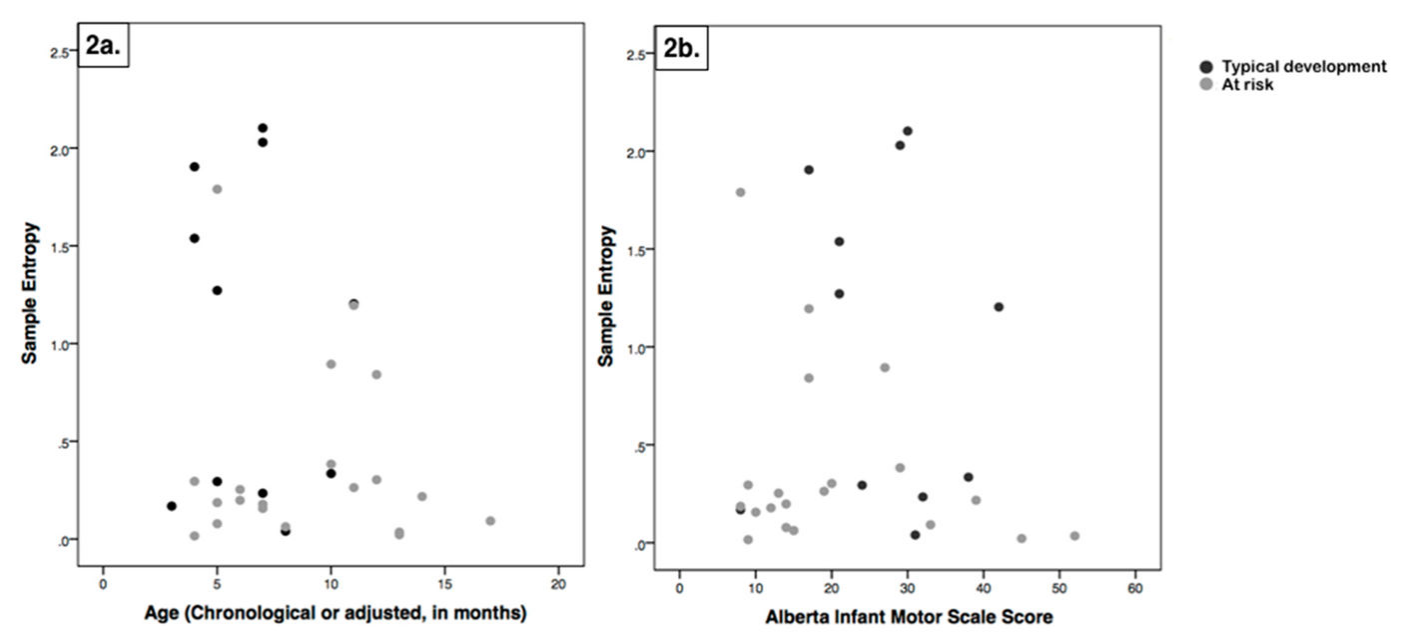
Sample entropy values for each infant by chronological or adjusted age in months (**a**) and by Alberta Infant Motor Scale scores (**b**). Infants with typical development are shown by black dots, while infants at risk are shown by gray dots.

SampEn median values and range of values are shown by group in Table 2. There was a significant group difference using Mann–Whitney (*p* = 0.02). The infants AR demonstrated smaller SampEn values than the infants with TD. There were not significant group differences in age (*p* = 0.07) or Alberta Infant Motor Scale scores (*p* = 0.8). SampEn median values for each infant are shown by age and Alberta Infant Motor Scale score in Figure 2.

## 4. Discussion

SampEn values of spontaneous leg movements were smaller in infants AR compared to infants with TD. Lower values of SampEn indicate more similarity in patterns across time, and may indicate more repetitive, less exploratory behavior in infants AR compared to infants with TD. This is consistent with findings from similar measures in other populations, for example spontaneous leg movements of infants with myelomeningocele were more regular and repeatable (lower Approximate Entropy values) than movements of infants with TD, indicating less adaptive and flexible movement patterns [30]. Similar findings have also been shown during supine lying in preterm infants [31], and during the development of independent sitting in infants with cerebral palsy [32] and infants with developmental delay [33]. Preterm infants and infants with cerebral palsy and developmental delay demonstrated more regular and repeatable center of pressure patterns (lower Approximate Entropy values) than infants with TD.

Our interpretation of our results is that lower values of SampEn indicate more similarity in patterns across time, and may indicate more repetitive, less exploratory behavior. It is possible that this behavior emerges as a result of reduced or altered neuromotor control that affects the interaction with the environment and the development of subsequent skills. This interpretation fits with the theoretical claim that variability in movement is necessary for exploration and precedes the emergence of a new behavior [34,35]. Higher values of SampEn in infants with TD could indicate that they have necessary exploration for supporting the emergence of new functional skills, while lower values in infants AR could indicate insufficient exploration and be related to developmental delay. Given the variability that can be observed in Figure 2, further investigation is necessary. It is possible that infants with higher SampEn values (either TD or AR) are on the cusp of emergence of a new functional skill, while infants with lower SampEn values are in a relatively more stable portion of their developmental trajectory. Higher SampEn values may indicate that a new skill is emerging for an infant with TD or AR; longitudinal assessment would be necessary to investigate this. We would hypothesize an increase in SampEn values of spontaneous leg movements prior to the emergence of a new functional skill. We would like to test this in a future study with longitudinal data collections clustered around the emergence of a new functional skill, such as walking onset.

Considering the Alberta Infant Motor Scale and SampEn results together, lower values in infants AR could indicate insufficient exploration and be related to developmental delay. This supports the need for effective early intervention, without waiting for infants to fall severely behind [36]. We would like to pursue an early intervention study aimed at increasing movement exploration and variability early in infancy, before infants have a chance to fall behind. This has been proposed in regard to intervention to promote sitting [37,38], and we propose it here in regard to promoting walking. Previous work has shown that treadmill stepping practice can promote the onset of walking in infants with Down syndrome [39], but a similar approach was not effective in infants born preterm [40]. It is possible that infants with Down syndrome, due to ligamentous laxity, exhibit higher amounts of flexibility and adaptability and less repeatability than is desirable [19], while other populations of infants (at risk of cerebral palsy, preterm) exhibit lower amounts of flexibility and adaptability and higher repeatability than is desirable. If this is true, then treadmill training would be sufficient for infants with Down syndrome, who do not need ‘more variability’, but not sufficient in most other populations, who would also need to experience ‘more variability’ in addition to practice stepping on a treadmill. This fits in with the notion of error-based learning that has been proposed for learning to walk in toddlers with cerebral palsy [41].

There are several limitations to this study. We had a heterogeneous sample of infants in regard to skill level, age, and risk level for developmental delay. Further, although we collected a full day of continuous leg movement data with wearable sensors, we only analyzed a short portion of it here. We did not assess reliability of SampEn values. Activities performed while playing were broad, and varied between infants and sometimes across samples from the same infant. For example, SampEn values from different samples from the same infant sometimes had apparently small intra-subject variability (range of 0.2), while other infants demonstrated apparently larger intra-subject variability (ranges of 0.7 or 1.7). We do not know if this is because the playing behavior was different, or because the measurement reliability is poor in certain conditions. Reliability would be best assessed with concurrent video data of movement behaviors and comparison of repeated SampEn data points from both similar and different behaviors. We chose to use multiple samples and median values in an effort to reflect more typical behavior and address this in our analyses here, however, given the variability in Figure 2, further investigation is warranted. Our goal here was to provide preliminary data to support our proposed approach and to provide pilot data for future, more tightly controlled studies. While Figure 2 shows that there are infants in both groups (TD and AR) with higher SampEn values, our statistical analyses supports that, in general, there are more TD than AR infants with higher SampEn values.

In summary, we propose that our preliminary work here supports that SampEn be pursued in future research as full-day wearable sensor data are collected from infants. Our initial results here support SampEn as a measure of variability of limb movement and a marker of the development of neuromotor control. We have demonstrated here that there are differences between infants with TD and AR, as measured from wearable sensor spontaneous leg movement data. In future research, we propose to relate SampEn to development of functional skills and developmental rate.

## Figures and Tables

**Table 1:** Participant Characteristics (Mean and Standard Deviation).

Group	Chronological or Adjusted Age (Months)	Alberta Infant Motor Scale
Typical Development	6.4 (2.5)	26.6 (9.7)
At Risk	9.0 (3.8)	20.5 (12.9)

**Table 2:** Sample Entropy Values by Group.

Group	Median	Range
Typical Development	1.20	0.40–2.10
At Risk	0.21	0.02–1.79
